# Our Contemporaries

**Published:** 1916-10

**Authors:** 


### OUR CONTEMPORARIES

The Medical Times, Aug., under the heading of Poliomyelitis, says ...... "If the Department of Health (of N. Y. State) ... had limited itself to what most of us consider its proper sphere, it would in all probability have been in a better position to cope with the epidemic or to prevent it. ...... The epidemic ... should do more to regulate the Department's aberrant activities than all the conventional and apparently useless forms of protest combined. ...Too much remains to be done in public health work of a legitimate character, as is now tragically and indubitably revealed, for the community further to tolerate doctrinaire philanderings, involving among other things the greasing of the wheels of attempted socialization." ....

We have sometimes felt that the forestry commissions might conserve to better purpose the raw material which private and public health authorities so copiously use to "release for publication" statements either already well known by the profession, or not known at all, or not susceptible of practical assimilation by the laity. We have also felt that such authorities sometimes go far afield from the proper work for which they are hired and that they sometimes claim undue publicity for the possession of knowledge which is the common property of the medical profession and for the application of this knowledge to practical ends, as almost any private physician might do if he had the proper backing. But we do not think that the State Board of Health deserves this scourging either on general principles or on account of not having prevented or checked the poliomyelitis epidemic.

Does anyone know just what Commissioner Biggs and his staff could have done to prevent the epidemic? Just what blunders have these men made in the management of the emergency with which they have been confronted?

---

The American Journal of Tropical Diseases, and Preventive Medicine has been absorbed by the New Orleans Medical and Surgical Journal. The latter is one of the oldest and best medical journals of the country, antedating the Buffalo Medical Journal by a few issues but slightly below us in number of annual volumes, probably on account of suspension during part of the Civil War. There are only two extant medical journals in the western continent established prior to 1845, the Boston Medical and Surgical Journal, and the American Journal of the Medical Sciences, originally a scientific rather than a medical publication. The N. Y. Medical Journal, however, really belongs in this group through the incorporation of the Medical News.

